# 

**Published:** 1886-02

**Authors:** 


PAGE.

**ORIGINAL COMMUNICATIONS:**

Antipyrin in Typhoid Fever. M. Mannheimer ---- 97

Opium-smoking. C. W. Earle ---- 104

Hydatiform Pregnancy. E. J. Doering ---- 112

Affections of the Skin induced by Cold Weather. J. N. Hyde ---- 116

Watery Discharges of Pregnant Women. C. W. Earle ---- 138

Hygienic Clothing. L. L. McArthur, 144

**SOCIETY REPORTS.**

*Gynecological Society.* Two Recent Models of Axis-Traction Forceps. W. W. Jaggard ---- 153

PAGE.

Pelvic Abscess. H. T. Byford ---- 160

*Chicago Medical Society, Meeting of January 4th.* Effects of Cocaine on the Central Nervous System. D. R. Brower ---- 173

Opium-smoking. C. W. Earle ---- 104

Hygienic Clothing. L. L. McArthur, 144

**BOOK REVIEWS:**

Consumption: Its Nature, etc. J. M. W. Kitchen ---- 188

Urinary and Renal Diseases. William Roberts ---- 189

**NEWS ITEMS** ---- 191

